# Supplementary material for: MicroRNA-like snoRNA-Derived RNAs (sdRNAs) Promote Castration-Resistant Prostate Cancer
Source: Cells. 2022 Apr 12;11(8):1302. doi: 10.3390/cells11081302 (PMC9032336; doi:10.3390/cells11081302)

Supplementary File S1. Select misannotated human microRNAs processed from snoRNAs.

Images generated by Ensembl genome browser. **(A)** Human microRNA hsa-miR-664b is entirely embedded in SNORA36A

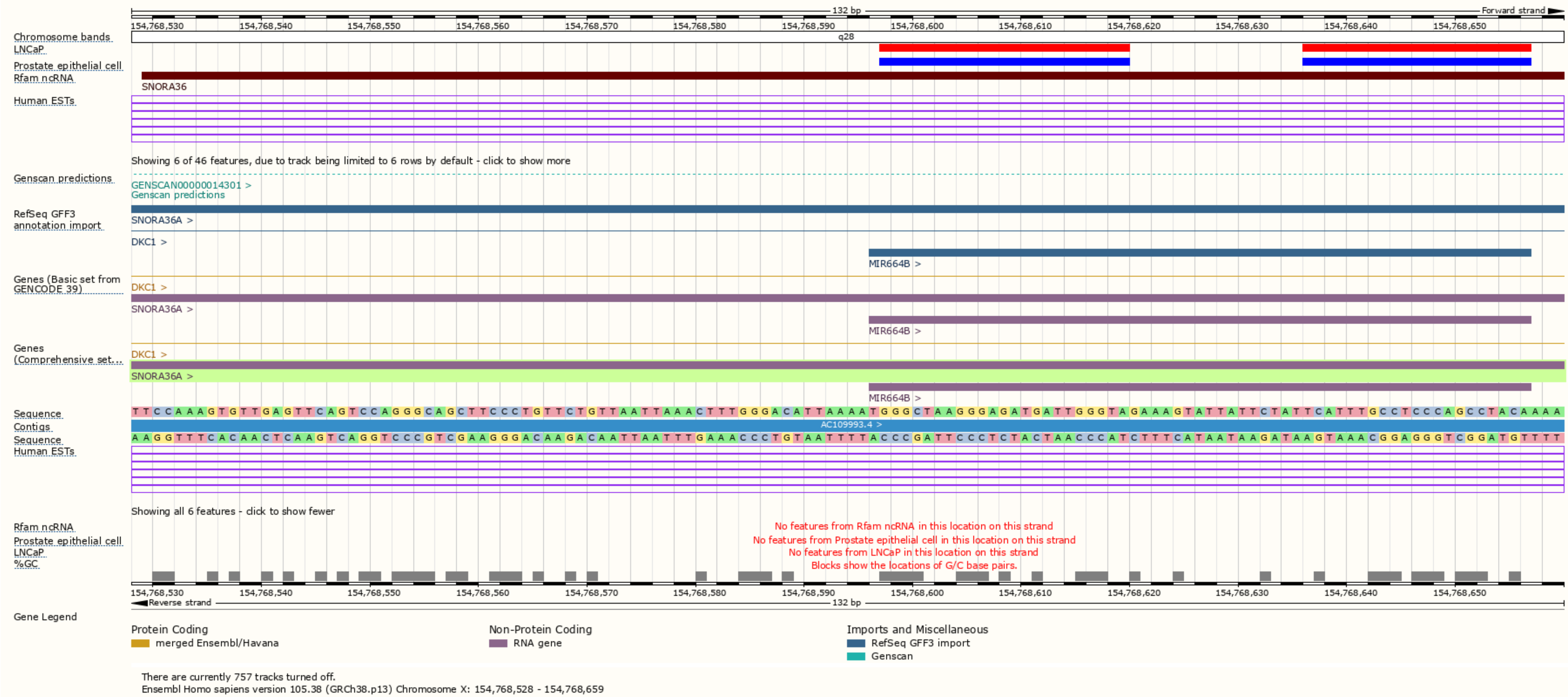

Supplementary File 1. Select misannotated human microRNAs processed from snoRNAs.

(B) Human microRNA hsa-miR-664a is entirely embedded in SNORA36B.

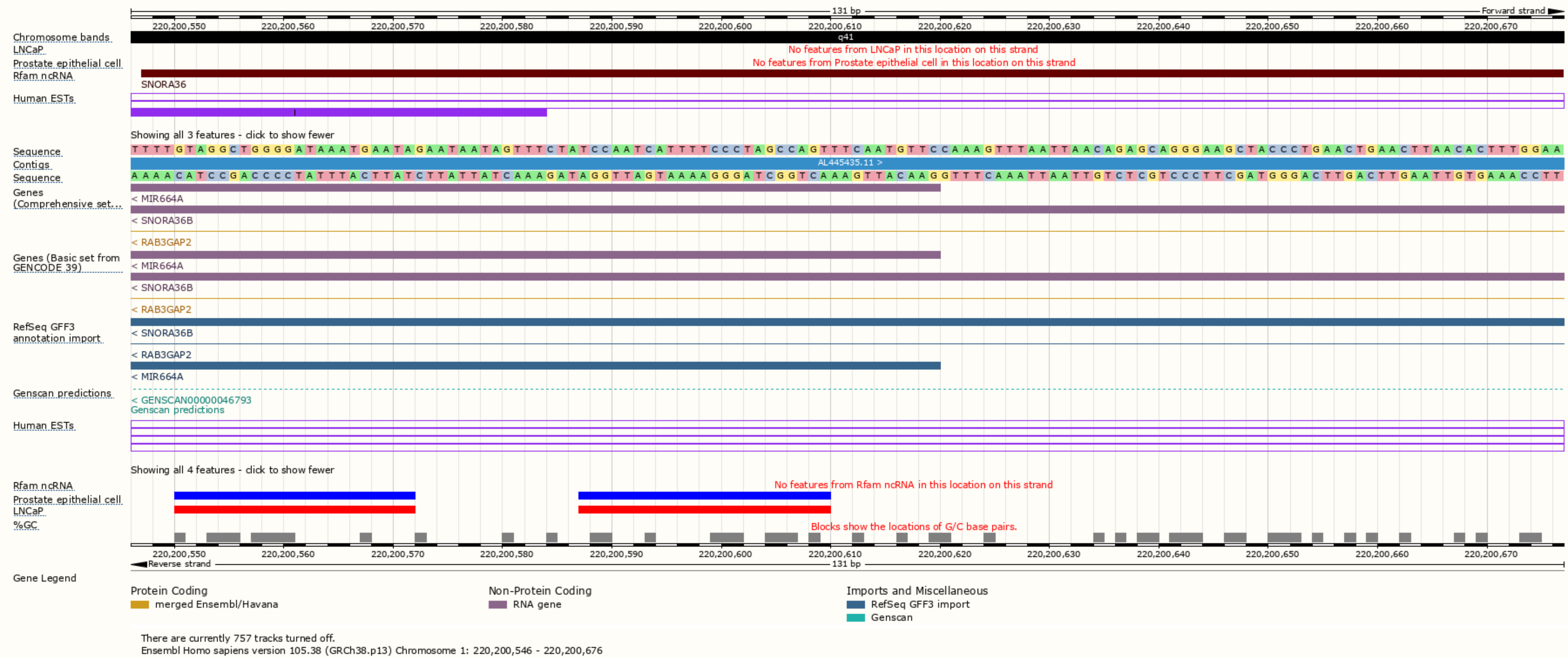

Supplementary File 1. Select misannotated human microRNAs processed from snoRNAs.

(C) Human microRNA hsa-miR-1291 is entirely embedded in SNORA2C.

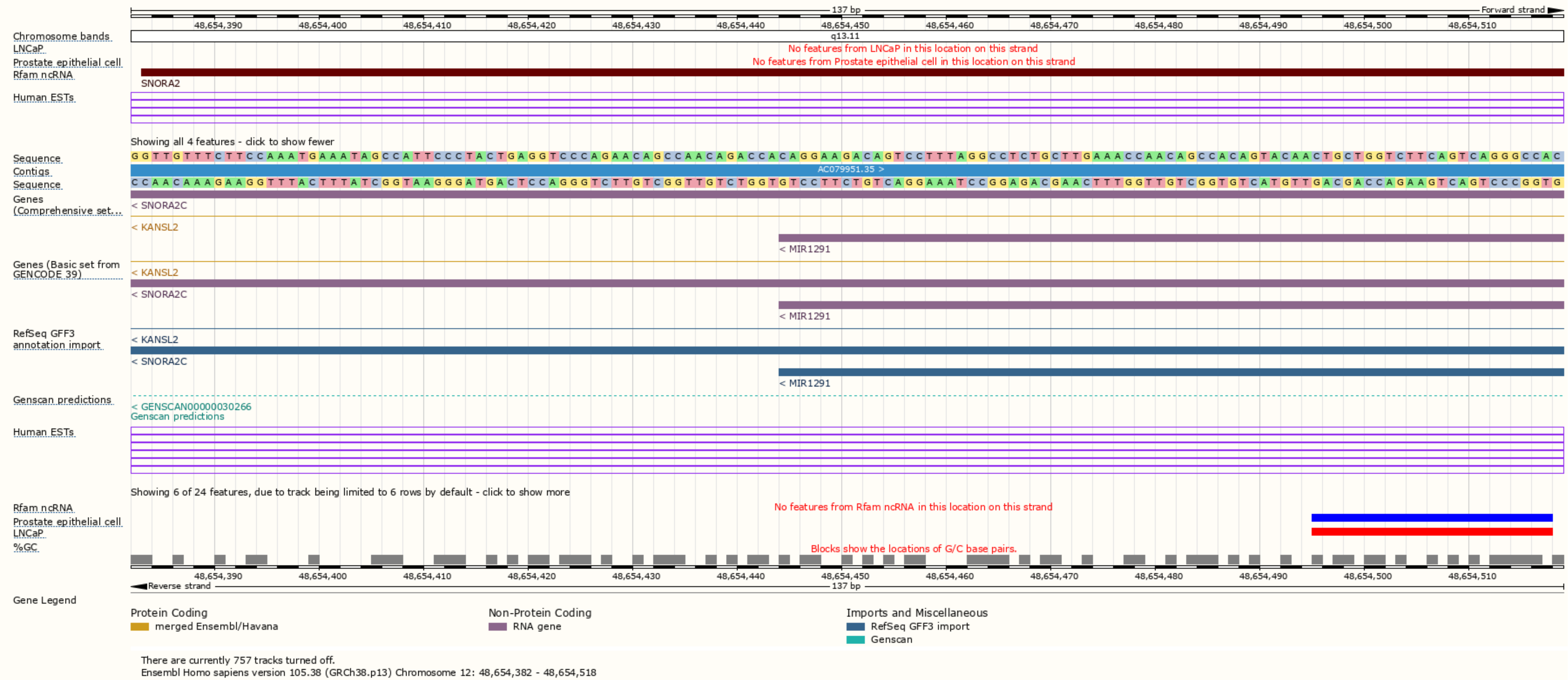

Supplementary File 1. Select misannotated human microRNAs processed from snoRNAs.

(D) Human microRNA hsa-miR-3651 is entirely embedded in SNORA84.

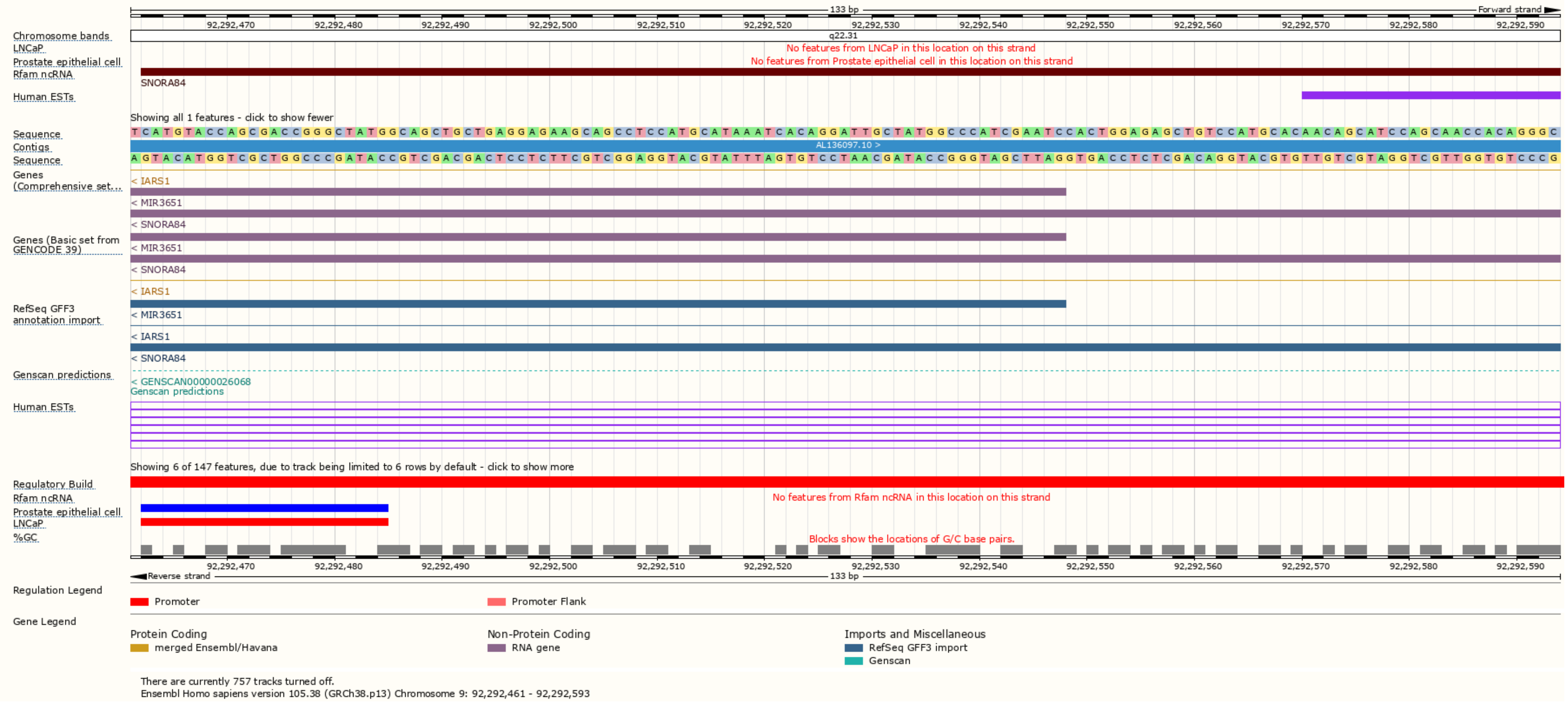

Supplementary File 1. Select misannotated human microRNAs processed from snoRNAs.
(E) Human microRNA hsa-miR-1248 is entirely embedded in SNORA81.

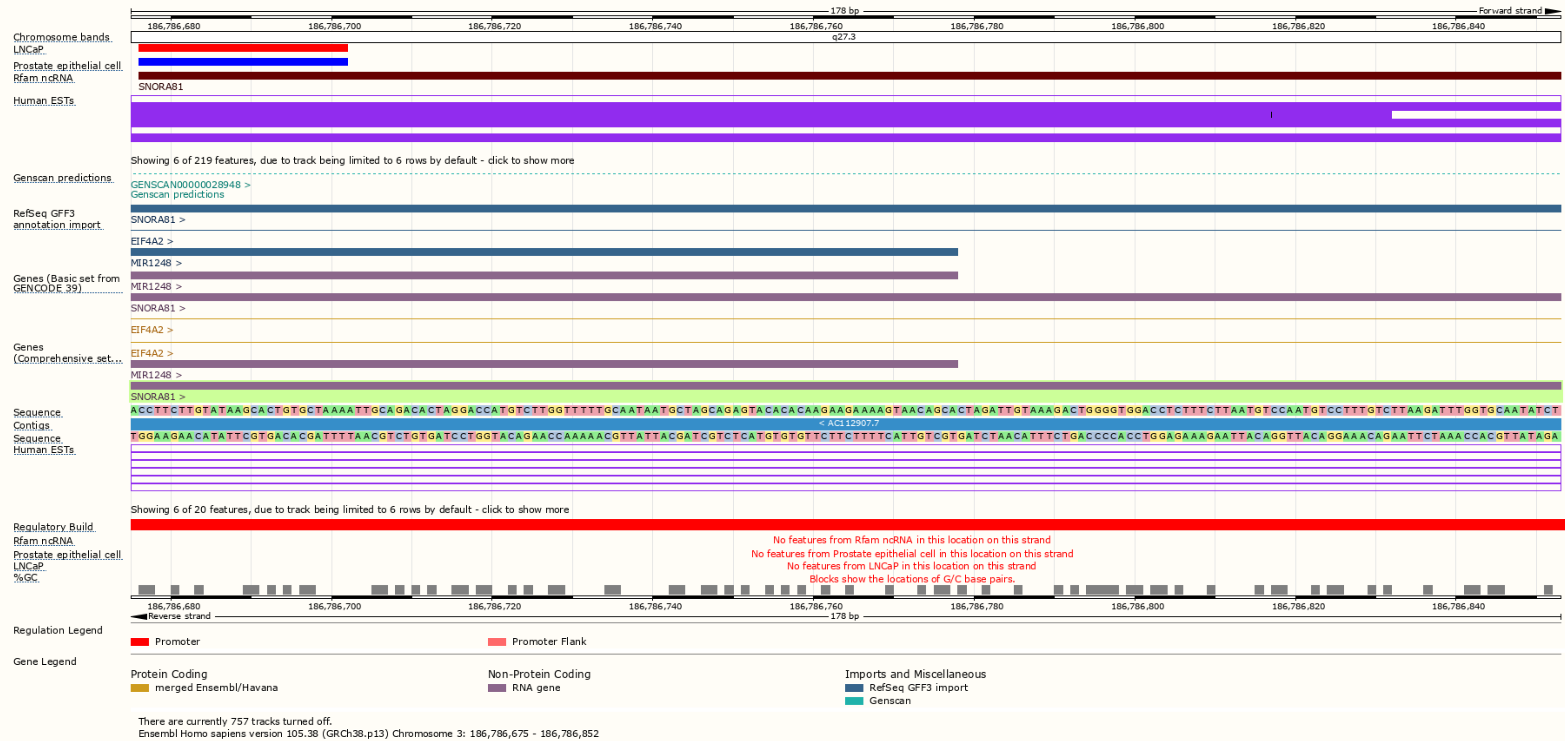

Supplement: Supplementary file 1 [file cells-11-01302-s001.zip › Supplementary File S1. Misannotated sdRNAs.pdf]
