# Supplementary material for: MicroRNA-like snoRNA-Derived RNAs (sdRNAs) Promote Castration-Resistant Prostate Cancer
Source: Cells. 2022 Apr 12;11(8):1302. doi: 10.3390/cells11081302 (PMC9032336; doi:10.3390/cells11081302)

Supplementary File S3. SdRNA -D19b and -A24 PC3 cell scratch assays. Representative migration (wound-healing) assays for PC3 cells transfected with the indicated sdRNA mimic or inhibitor. **(A)** 19 inhibitor.

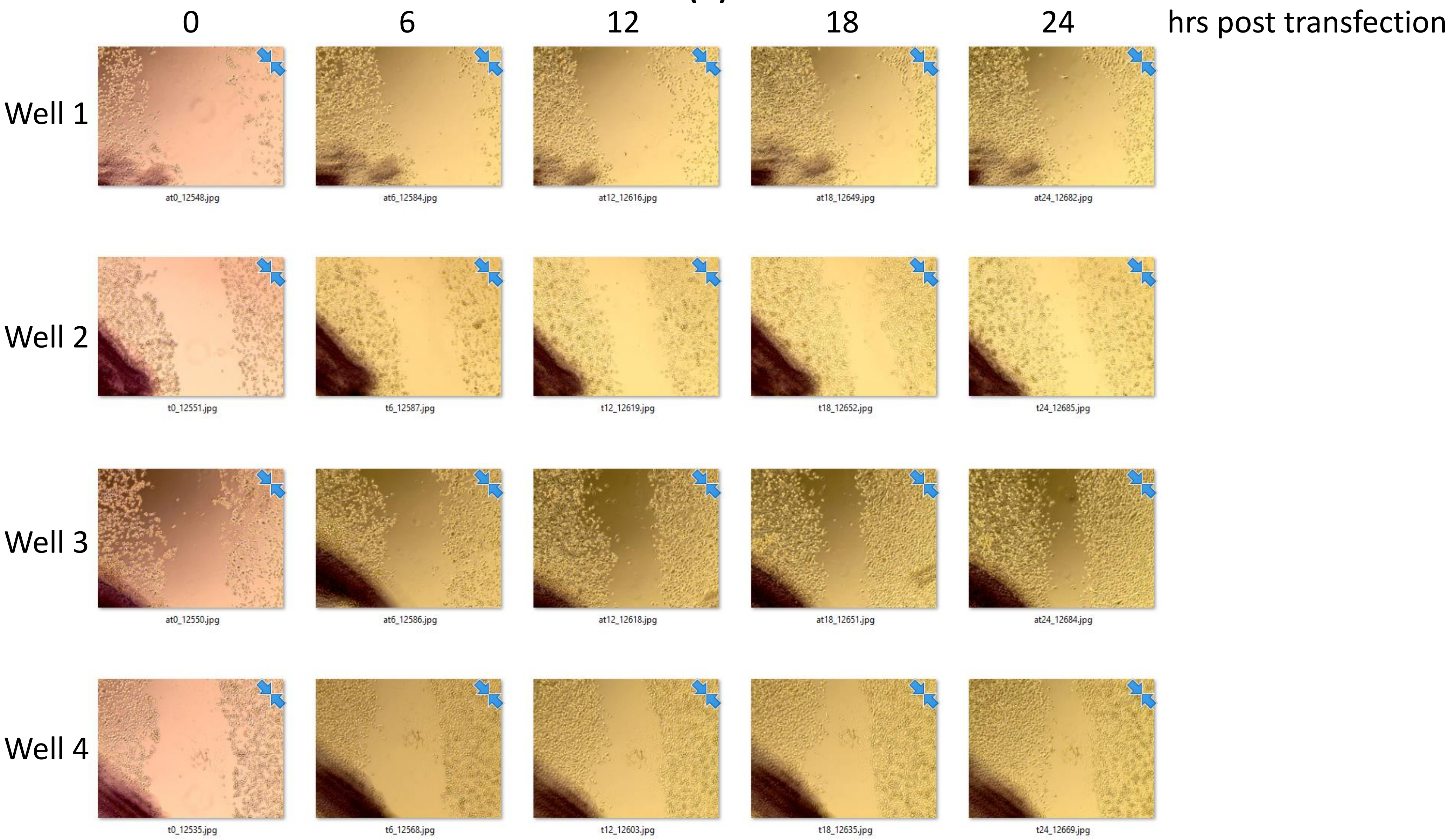

Supplementary File 3. SdRNA -D19b and -A24 PC3 cell scratch assays. (B) 19 mimic.

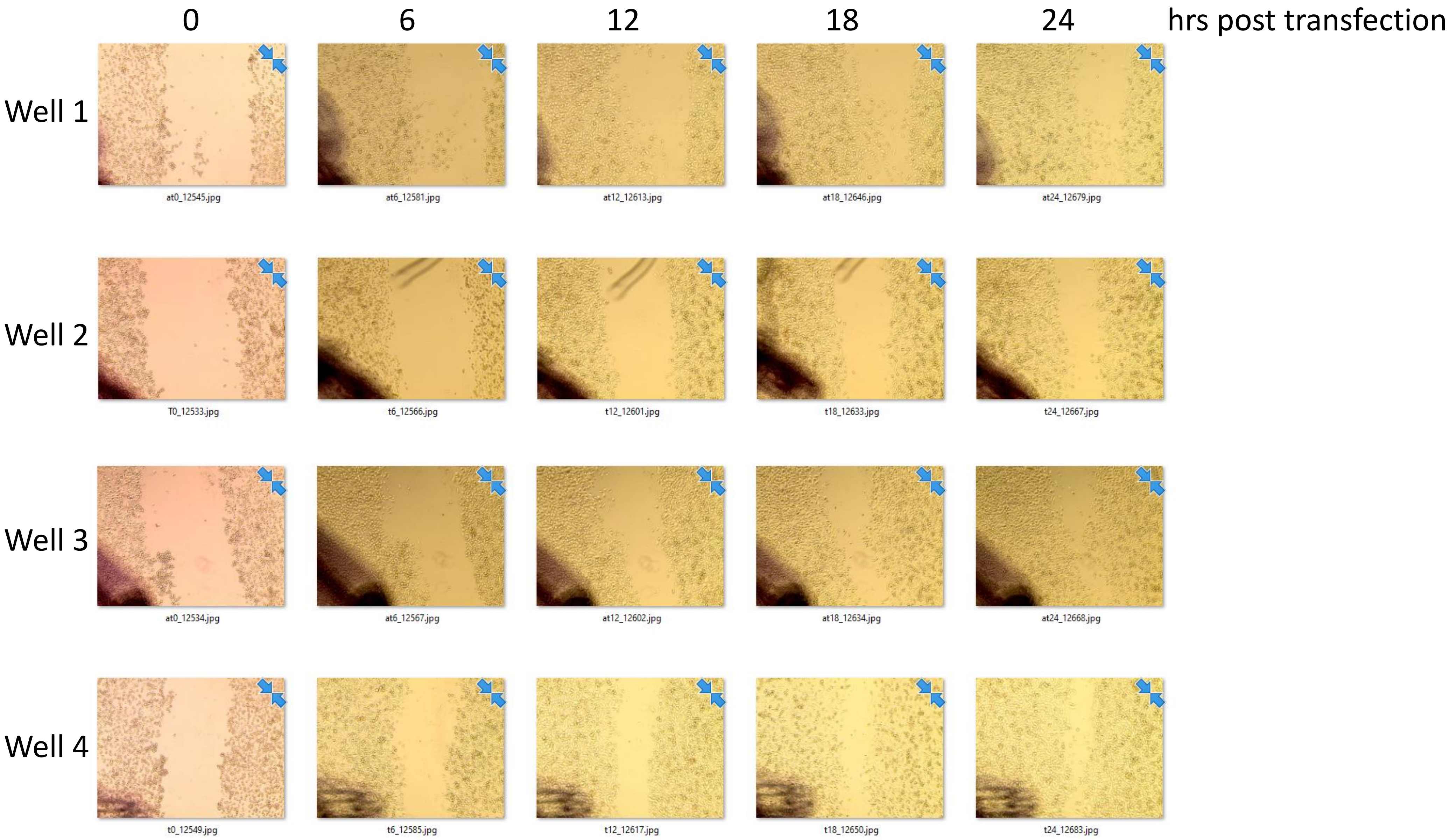

Supplementary File 3. SdRNA -D19b and -A24 PC3 cell scratch assays. (C) 24 inhibitor.

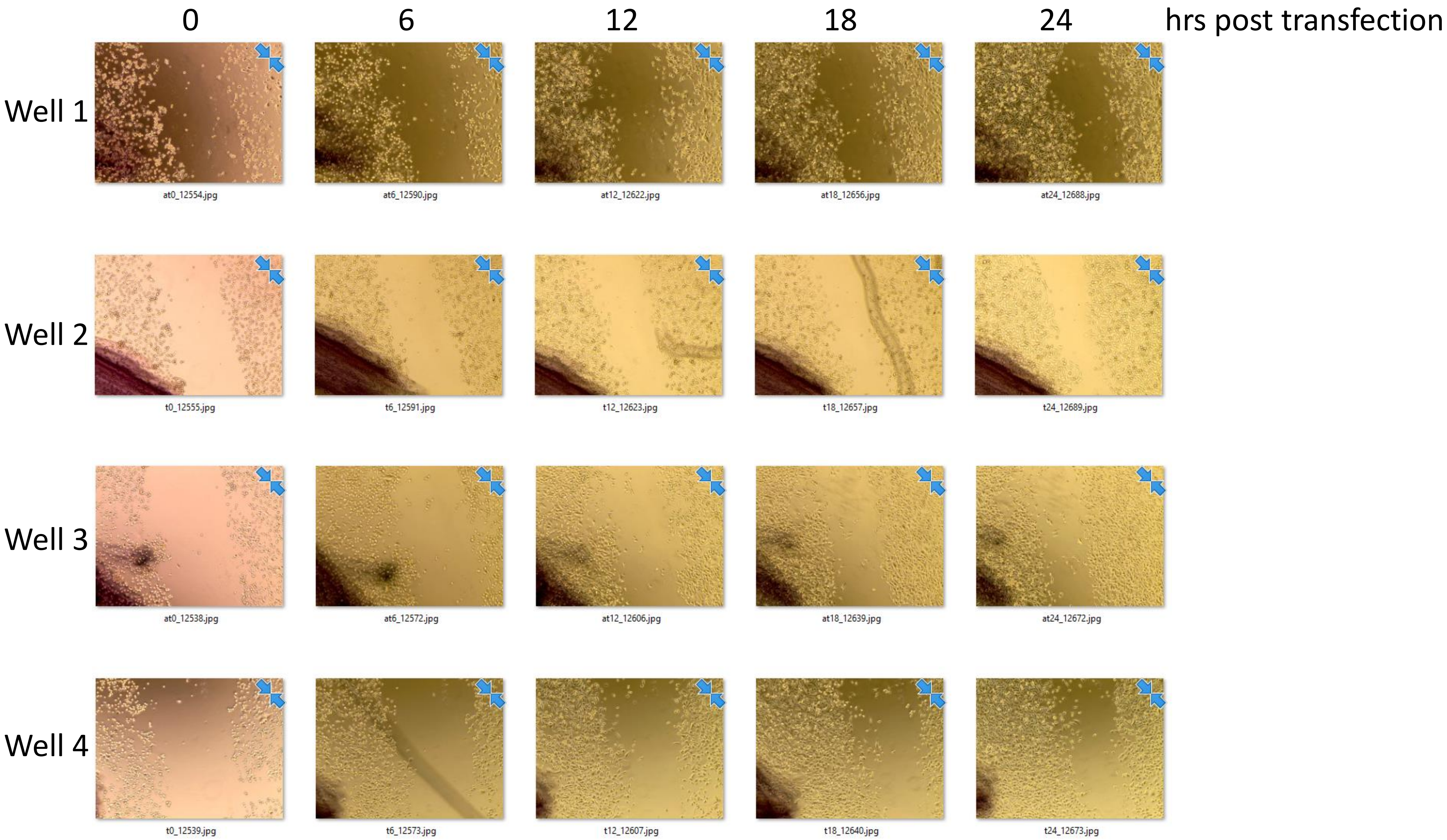

Supplementary File 3. SdRNA -D19b and -A24 PC3 cell scratch assays. (D) 24 mimic.

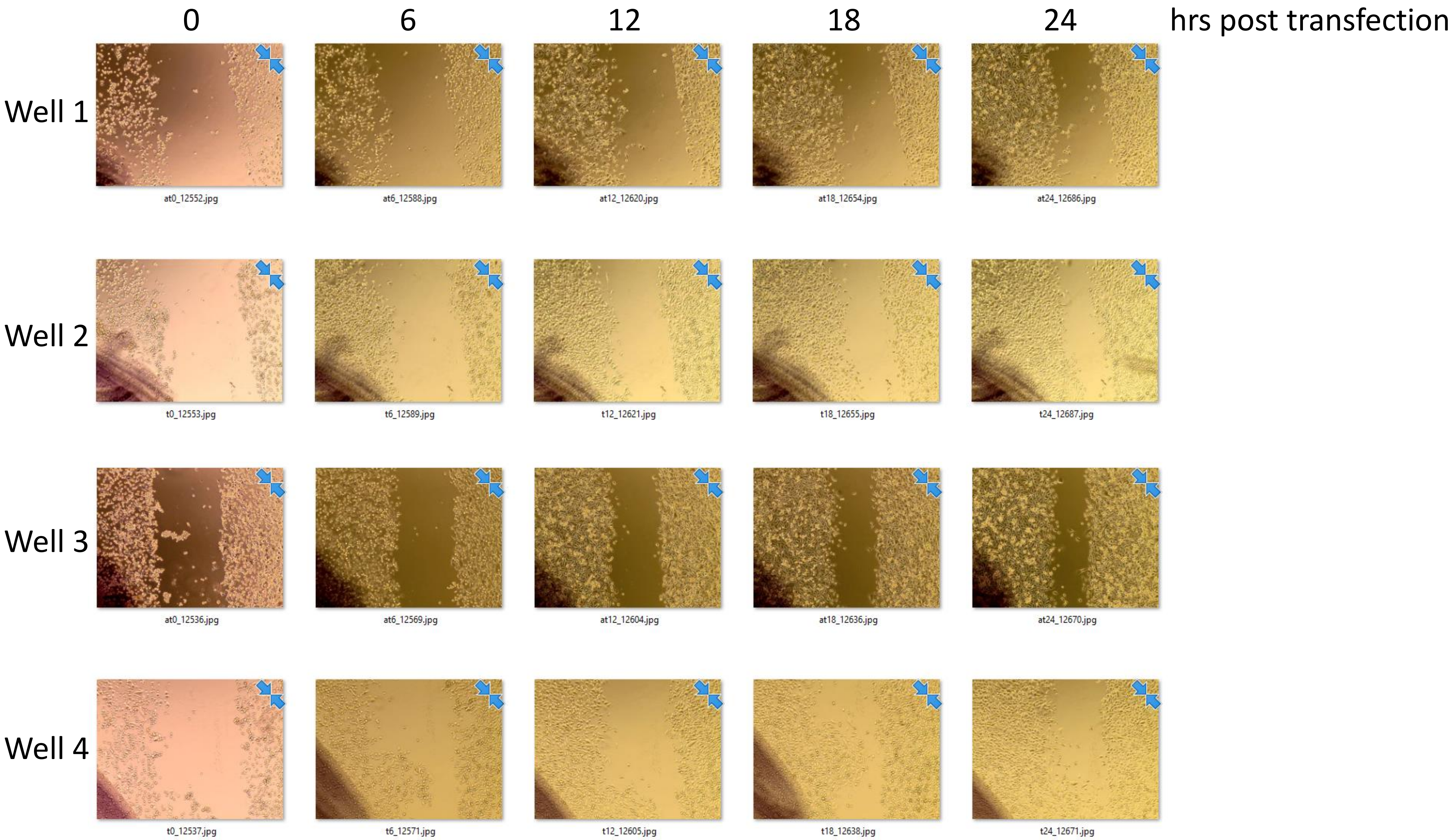

Supplementary File 3. SdRNA -D19b and -A24 PC3 cell scratch assays. (E) 42 inhibitor.

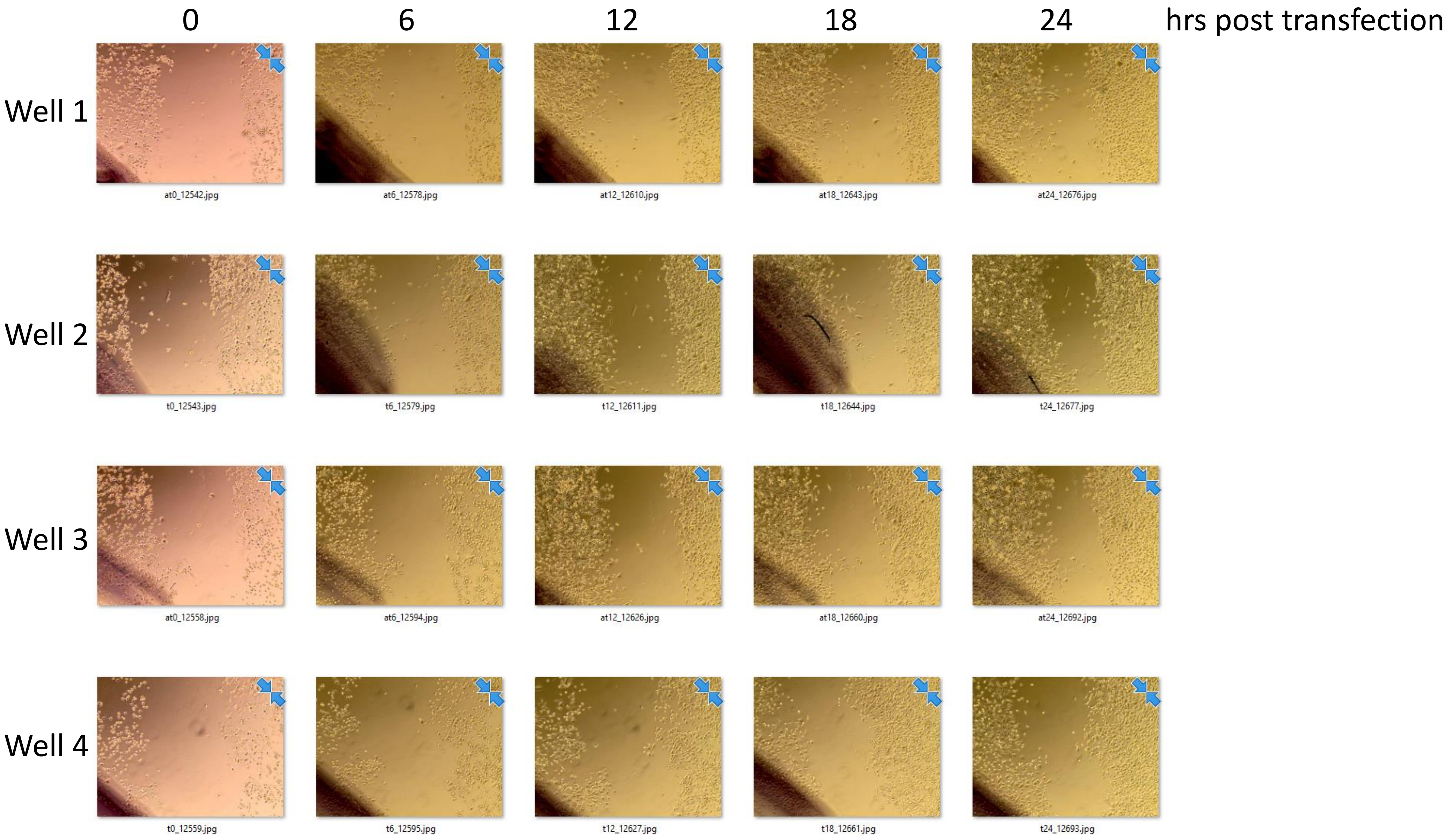

Supplementary File 3. SdRNA -D19b and -A24 PC3 cell scratch assays. (F) 42 mimic.

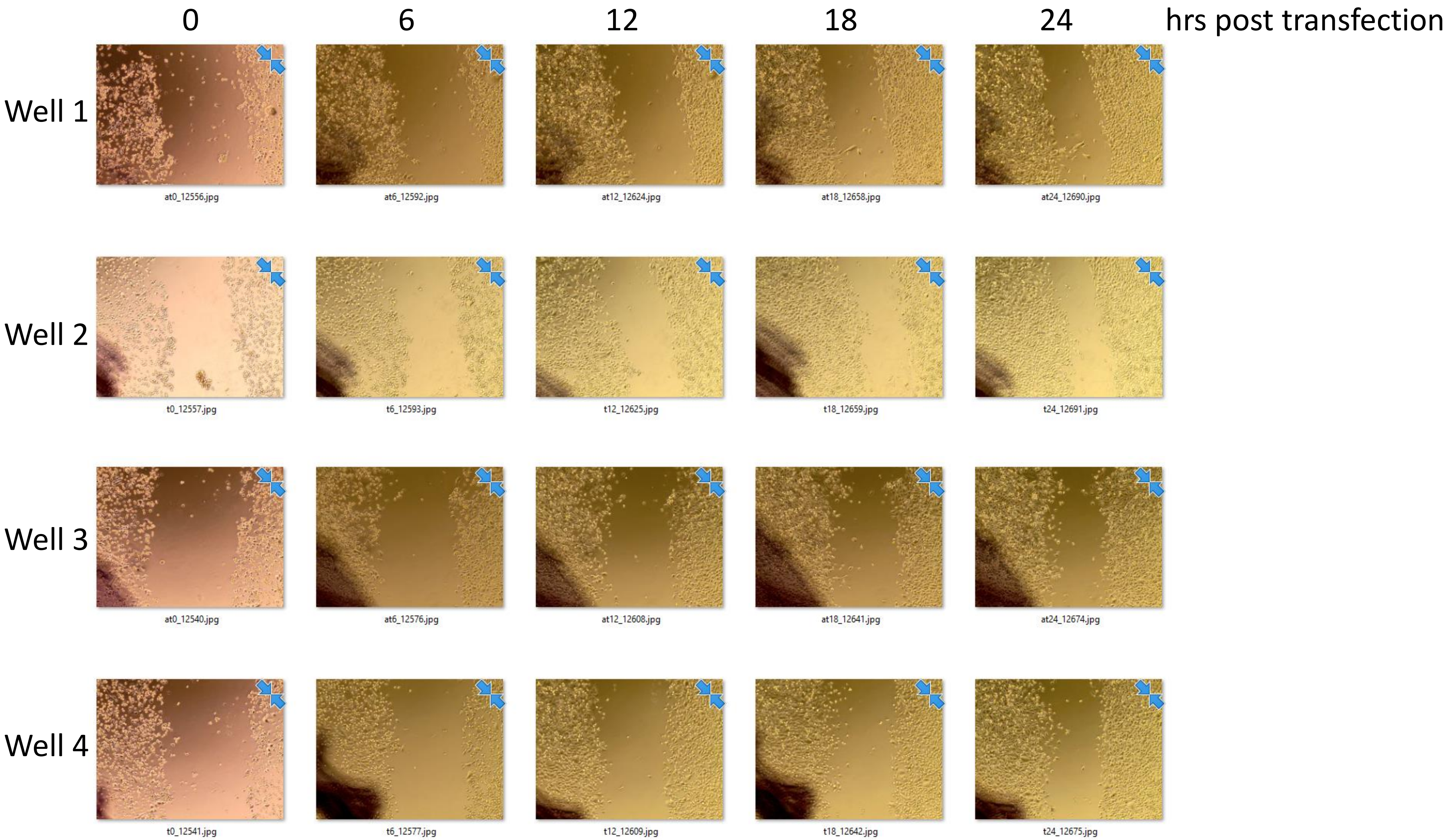

Supplementary File 3. SdRNA -D19b and -A24 PC3 cell scratch assays. (G) Control inhibitor.

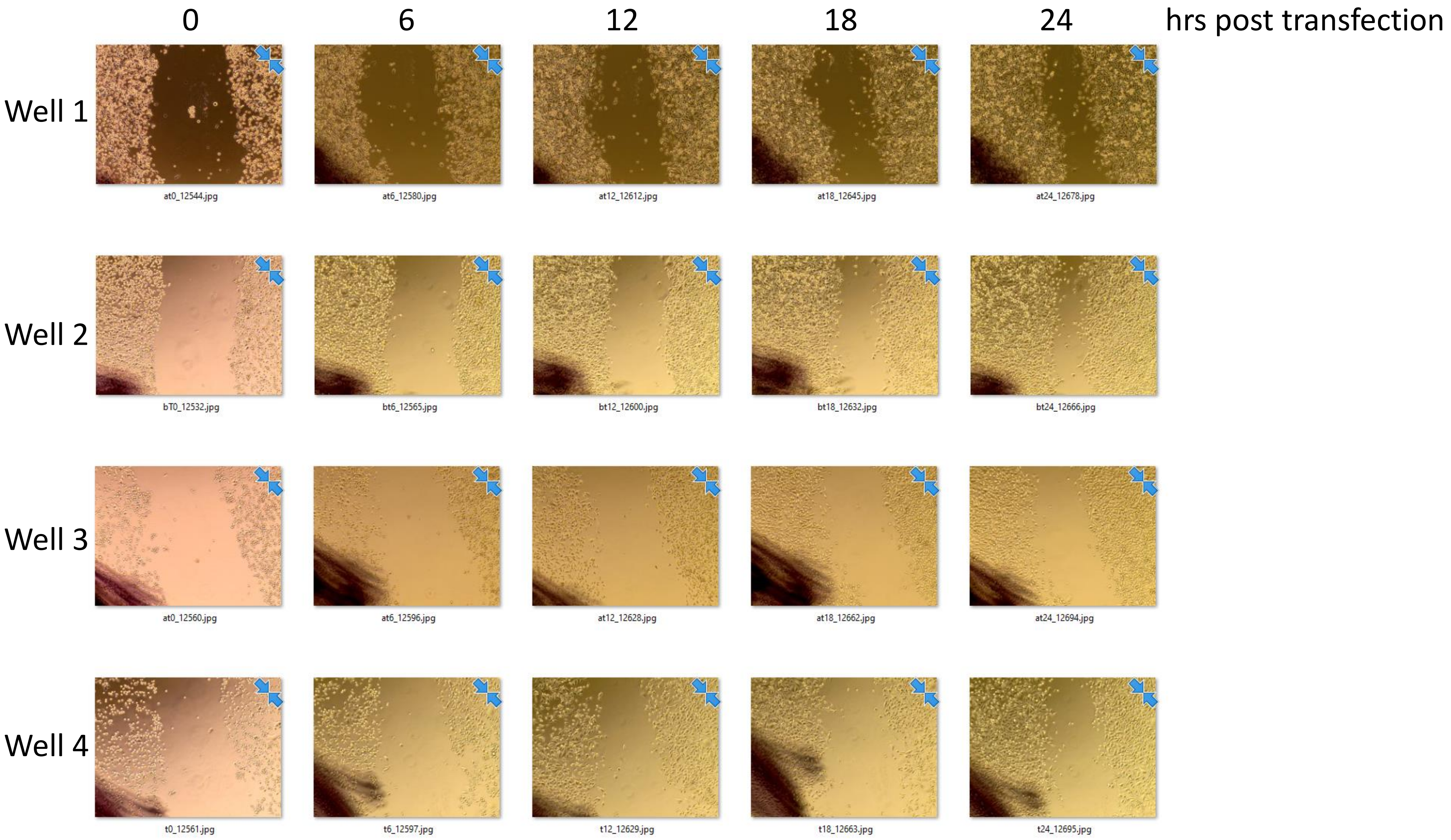

Supplementary File 3. SdRNA -D19b and -A24 PC3 cell scratch assays. (H) Control mimic.

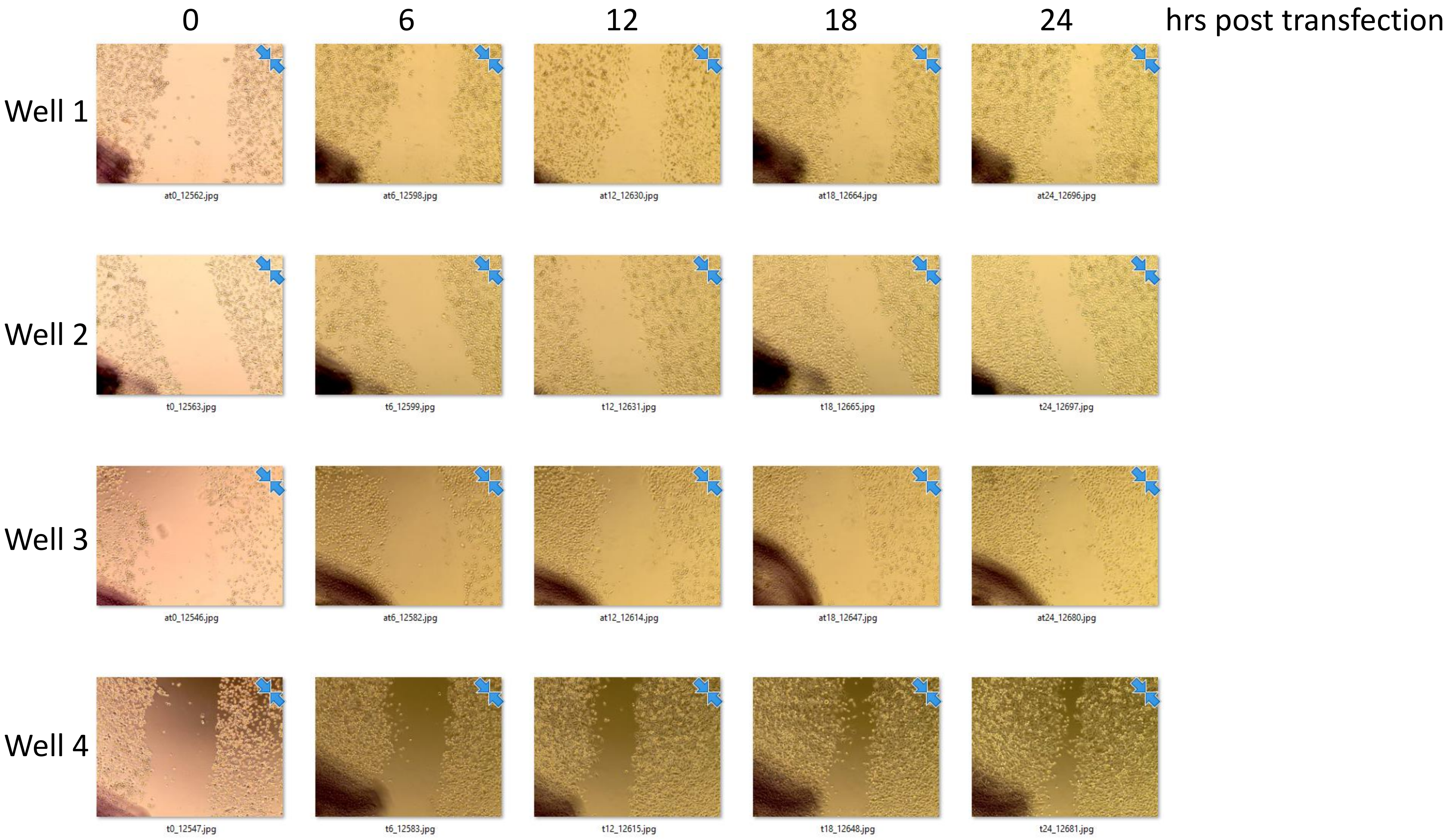

Supplementary File 3. SdRNA -D19b and -A24 PC3 cell scratch assays. (I) Mock control.

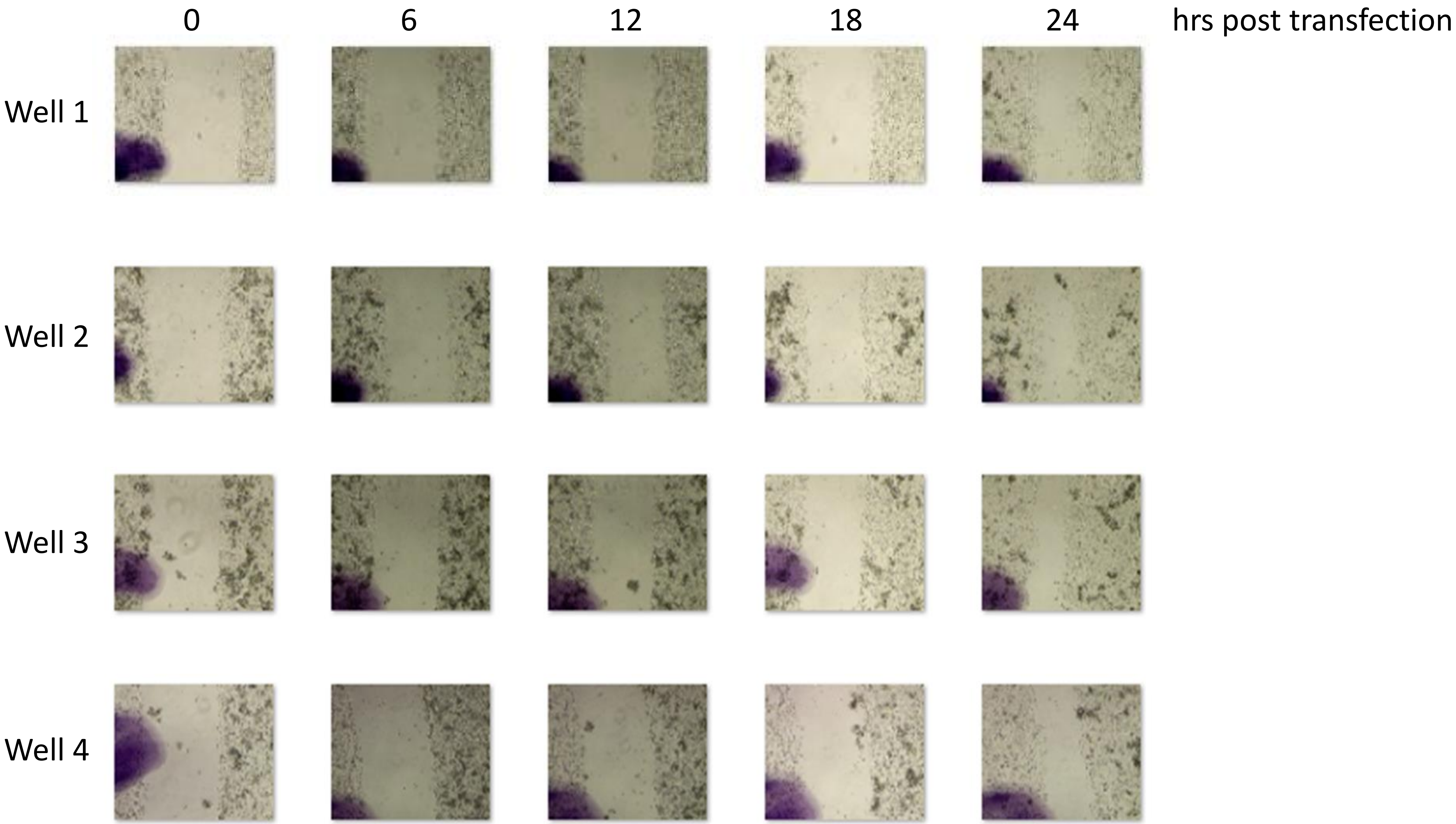

Supplement: Supplementary file 1 [file cells-11-01302-s001.zip › Supplementary File S3. PC3 scratch assays NEW.pdf]
