# Supplementary material for: MicroRNA-like snoRNA-Derived RNAs (sdRNAs) Promote Castration-Resistant Prostate Cancer
Source: Cells. 2022 Apr 12;11(8):1302. doi: 10.3390/cells11081302 (PMC9032336; doi:10.3390/cells11081302)
Supplement: Supplementary file 1 [file cells-11-01302-s001.zip › Supplementary File S5. Oligonucleotide master list.pdf]

Supplementary File S5. Oligonucleotide master list. All sequences listed 5' to 3'.

Mimics were ordered as custom miRIDIAN miRNA mimics (Horizon Discovery) at 0.015 µmol scale. miRIDIAN Mimics are double-stranded RNA oligonucleotides chemically enhanced with the ON-TARGET modification pattern to preferentially program RISC with the active microRNA strand.

sd_A24	Active strand	P-CUCCAUGUAUCUUUGGGACCUGUCA
	Passenger	ACAGGUCCCAAAGAUACAUGGAGUU
sd_D19b	Active strand	P-AUUACAAGAUCCAACUCUGAU
	Passenger	CAGAGUUGGAUCUUGUAAUUU
sd_D58c	Active strand	P-UUGCUGUGAUGACUAUCUUAGGAC
	Passenger	CCUAAGAUAGUCAUCACAGCAAUU
sd_D42A	Active strand	P-CAUGAACAAAGGAACCACUGAA
	Passenger	CAGUGGUUCCUUUGUUCAUGUU
sd_D24	Active strand	P-CUAUCUGAGAGAUGGUGAUGAC
	Passenger	CAUCACCAUCUCUCAGAUAGUU
sd_Ctl	Active strand	P-UAAUCUGACCAAGACUCUUAA
	Passenger	AAGAGUCUUGGUCAGAUUAUU

Inhibitors were ordered as custom IDT miRNA Inhibitors (Integrated DNA Technologies) at 5 nmol scale. IDT miRNA Inhibitors are RNA oligonucleotides comprised of 2'-O-methyl residues that confer increased binding affinity to RNA targets and resistance to endonuclease degradation. ZEN modifications are included to block exonuclease degradation.

sdRNA-D19b	mA/ZEN/mUmCmAmGmAmGmUmUmGmGmAmUmCmUmUmGmUmAmA/3ZEN/
sdRNA-A24	mA/ZEN/mCmAmGmGmUmCmCmCmAmAmAmGmAmUmAmCmAmUmGmGmAmG/3ZEN/
sdRNA-A42	mC/ZEN/mAmGmUmGmGmUmUmCmCmUmUmUmGmUmUmCmAmUmG/3ZEN/
sdRNA-A61	mC/ZEN/mCmUmGmUmCmUmGmAmAmAmCmUmAmGmCmCmCmAmCmAmU/3ZEN/
sdRNA-93	mA/ZEN/mGmAmGmUmUmCmUmCmAmUmCmCmUmUmGmGmCmCmA/3ZEN/
Scram Ctl	mA/ZEN/mAmGmAmGmUmCmUmUmGmGmUmCmAmGmAmUmUmA/3ZEN/

Primers were ordered as custom DNA oligos (Integrated DNA Technologies) at 25 nmol scale.

CD44 3'UTR TS Forward	ACTCGAGACCAAAGGTTTTCCATCCTGTCC
CD44 3'UTR TS Reverse	AGCGGCCGCACATCTCTCCTTTAAAGATATTCTAC
CDK12 3'UTR TS Forward	ACTCGAGAcaaaaacctttcaaacagagc
CDK12 3'UTR TS Reverse	AGCGGCCGCActgttcttcctcacagtatgc
sdRNA-D19b qRT-PCR	GCCCATTACAAGATCCAACTCTGAT
sdRNA-A24 qRT-PCR	GCTCCATGTATCTTTGGGACCTGTCA
U6 Forward	GCTCGCTTCGGCAGCACATATAC
U6 Reverse	CGCTTCACGAATTTGCGTGTCATCC
